# Fast alignment of reads to a variation graph with application to SNP detection

**DOI:** 10.1515/jib-2021-0032

**Published:** 2021-11-16

**Authors:** Maurilio Monsu, Matteo Comin

**Affiliations:** Department of Information Engineering, University of Padua, Padua 35100, Italy

**Keywords:** reads alignment, SNP detection, variation graph

## Abstract

Sequencing technologies has provided the basis of most modern genome sequencing studies due to its high base-level accuracy and relatively low cost. One of the most demanding step is mapping reads to the human reference genome. The reliance on a single reference human genome could introduce substantial biases in downstream analyses. Pangenomic graph reference representations offer an attractive approach for storing genetic variations. Moreover, it is possible to include known variants in the reference in order to make read mapping, variant calling, and genotyping variant-aware. Only recently a framework for variation graphs, *vg* [Garrison E, Adam MN, Siren J, et al. Variation graph toolkit improves read mapping by representing genetic variation in the reference. Nat Biotechnol 2018;36:875–9], have improved variation-aware alignment and variant calling in general. The major bottleneck of *vg* is its high cost of reads mapping to a variation graph. In this paper we study the problem of SNP calling on a variation graph and we present a fast reads alignment tool, named VG SNP-Aware. VG SNP-Aware is able align reads exactly to a variation graph and detect SNPs based on these aligned reads. The results show that VG SNP-Aware can efficiently map reads to a variation graph with a speedup of 40× with respect to *vg* and similar accuracy on SNPs detection.

## 1 Introduction

Advances in sequencing technologies and computational methods have enabled rapid and accurate identification of genetic variants in the human population. Although massive sequencing projects over the past decade such as the 1000 Genomes Project [1] have generated trillions of reads available from public archives [2], the ability to make use of these enormous datasets is still limited. One important limitation is that most analyses rely on the alignment of sequencing reads to the human reference genome, which does not reflect the genetic diversity of individuals or populations [3].

The reliance on a single reference human genome could introduce substantial biases in downstream analyses and result in an inability to detect disease related genetic variants [4, 5]. In fact, sequences from humans, specifically those not included in the samples used for constructing the human reference, may align incorrectly or not at all when they originate from a region that differs from the reference genome.

Accurate genotype calls and allele frequency estimations are crucial for population genomics analyses. In [3] the authors found a bias toward overestimation of the reference allele frequency for the 1000 Genomes Project [1] data, indicating mapping bias is an important cause of error in frequency estimation. Another example is the analysis of ancient DNA. The typical workflow for ancient DNA data processing starts with the alignment of sequencing reads to a linear reference genome, which contains only the reference allele at polymorphic sites. Reads containing the alternate allele are less likely to map than reads containing the reference allele, creating a potentially strong bias against non-reference variation, which can have a significant effect on population genetic inference and implications for many ancient DNA studies [6, 7].

A series of large-scale projects have characterized more than 660 million single-nucleotide polymorphisms (SNPs; in dbSNP [8]). Although these variants represent a valuable resource for genetic analyses, traditional alignment-based tools do not adequately incorporate them.

Pangenomic graph reference representations offer an attractive approach for storing genetic variation of all types [9]. These graphical data structures can seamlessly represent both structural variants and SNPs using the same graph. Moreover, including known variants in the reference makes read mapping, variant calling, and genotyping variant-aware. This leads to benefits in terms of accuracy and sensitivity [10, 11]. This model allows different variant types to be called and scored simultaneously in a unified framework. *vg* is the first openly available variation graph tool to scale to multi-gigabase genomes. It provides read mapping, variant calling, and visualization tools [10].

Aligning a sequence to a sequence is a well-studied problem with many highly optimized tools [12, 13]. In contrast, aligning sequences to graphs is a newer field and practical tools only start to emerge, where most of the existing tools are specialized for one purpose such as error correction [14], or hybrid genome assembly [15]. The *vg* toolkit [10] provides a set of general-purpose tools to work with genome graphs.

*vg* toolkit [10] improves genotyping by representing genetic variation in the reference. Variation graphs are bidirected DNA sequence graphs that compactly represent genetic variations across a population. Thanks to this, *vg* is able to map reads into arbitrary variation graphs using generalised compressed suffix arrays [16]. *vg* performs the alignment using graphs but, unfortunately, it uses a lot of time and memory, especially for the construction of graphs.

In this paper we study the problem of SNPs detection using a variation graph. We present an improvement of *vg*, named VG SNP-Aware, with the aim of boosting the most demanding phase of reads to graph alignment. It is possible to reduce the mapping time used by *vg* by considering only exact matches and using a depth-limited like search on the graph. VG SNP-Aware is able to significantly reduce the time of the alignment phase, while ensuring high precision values on SNPs detection.

## 2 Methods

### 2.1 Introduction to variation graph

A variation graph is used to represent multiple sequences, coming from different reference genomes, to cover-up all possible known variants. The goal is to have a sequence graph together with a set of paths, representing all possible sequences from a population.

The structure is defined by

**nodes**, containing the sequence;
**edges**, connecting two nodes via either of their respective ends;
**paths**, representing the possible sequences from a population.

Given a graph *G* = (*V*, *E*), it consists of a finite set of nodes *V* and a set of edges *E* ⊆ *V* × *V*. Each node *v*_*i*_ represents a sequence from the alphabet $\left\{A,C,G,T,N\right\}$ . Edges are directed and (*u*, *v*) ∈ *E* is an edge from node *u* to node *v*. The in-degree of a node is the number of incoming edges whereas the out-degree of a node is the number of outgoing ones. Let *P* = *v*_*o*_…*v*_*P*−1_ be a string over a set of nodes *V*, we denote by *P* a path in the graph *G* = (*V*, *E*), if all the edges of the path are in *E*. Graphs are bidirectional and each node has two orientations, each one representing a DNA strand. Nodes can be traversed in either forward or reverse direction.

A position in the graph is defined by the node ID, the direction and an offset with respect to the sequence represented by the node. Nodes are stored by ID, and the offset indicates the position of a single nucleotide on the sequence represented by the node. The offset counts from the start of the node in the specified orientation.

To enable read mapping and other random access operations on large graphs, in addition to the variation graph, *vg* uses a succinct representation of the variation graph (XG) and a Generalized Compressed Suffix Array (GCSA) index [17]. With the XG index is possible to get the distance between two nodes, to get adjacent nodes or to find the in degree of a node. The GCSA2 index allows quick lookups of where a specific sequence is in the graph. GCSA2 is a custom re-implementation of the Generalized Compressed Suffix Array (GCSA) [16]. GCSA2 approximates the graph with a de Bruijn graph, allowing it to index more complex graphs. The de Bruijn graph in GCSA2 is pruned (compressed structurally) by using strings shorter than *k* = 32 characters as nodes, if the shorter strings identify the start nodes of the corresponding paths uniquely. The pruned graph is encoded with a generalization of the FM-index. The index includes also extensions based on suffix trees.

The GCSA2 index supports the followings queries:

**rc()**, return true if a node is into the reverse complement strand, false otherwise;
**offset()**, return the offset of the node of the original graph;
**id()**, return the identifier of the node of the original graph.

Thanks to the GCSA2 queries it is possible to obtain the location of sequences on the variation graph, in linear time.

*vg* uses the “seeding and extending” technique to align reads to the graph. The alignment process is based on maximal exact match sequences between the query read and the reference graph. The *vg* mapping starts by finding seed matches using the GCSA2 indexes, then it clusters them (in case of multiple seeds close together) and, finally, it performs a local constrained dynamic programming alignment of the read on a region of the graph around each cluster. More details can be found in [10]. The major bottleneck of *vg* is the amount of computing time and memory required for reads mapping. In the following we will see how SNPs can simplify the variation graph and the alignment of reads to it.

### 2.2 SNPs analysis on a variation graph

**Figure 1:**
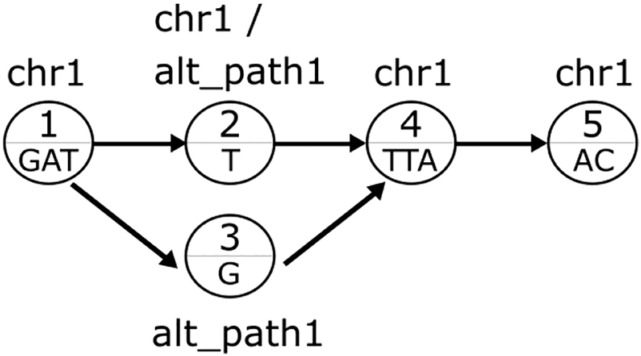
Example of a variation graph with a biallelic SNP. The two alternative bases are included in two distinct paths.

In Figure 1 we show an example of a variation graph and its structure for a single-nucleotide polymorphism. *vg* represents the two nucleotides of a biallelic SNP as two possible nodes on graph and for each of them provides an edge from and to it. This approach allows to have multiple paths which admits all possible sequences on graph, including the reference base or the alternative base of SNPs. The naming convention applied to the path requires alternative path having a name starting with “alt_”, this allows to distinguish it from the reference paths.

It is important to note that each node belongs to one or more paths. *vg* creates a path for each chromosome and for each variant. Thus, node with ID 1, is part of chr1 path. Node “2”, since it contains the reference nucleotide, contains both the chr1 path and the variant path (alt_path1), whereas node “3” belongs only to the alt_path1, since it contains the alternative nucleotide. The construction of a variation graph starts from a reference genome and a set of set of SNPs. In this specific case, where only SNPs are considered, it is possible to study the properties of node IDs in the graph in the following way. If we consider the forward direction, the resulting node IDs are increasing, otherwise they are decreasing. This is due to the fact that nodes creation is sequential into the forward direction. In Figure 1 we show an example of consecutive node IDs on the graph. Unlike *vg*, this graph does not contains cycles or arcs between distant nodes that aim to reuse one or more nodes in the graph. These two properties, are due to the fact that we exploit the graph only in case of SNPs. This allows VG SNP-Aware to follow up the consecutive nature of node IDs for the alignment of reads.

### 2.3 VG SNP-Aware: variation graph optimized for SNP detection

VG SNP-Aware introduces an optimised alignment process for SNPs, with the idea to exploit only exact alignments, using a depth-limited search like. The process is composed by two distinct phases: searching for initial node and mapping from it. Both phases are based on the characteristics of the variation graph in case of SNPs. In the following we describe these two steps.

Given a read, in the first phase, the algorithm searches for one or more initial nodes to be used as seed for the alignment. The initial node is a node in the graph that shares at least 2 characters with the reads. Since by construction the string in a node is limited by *k* = 32 characters, also the initial alignment follow the same constraint. The research is carried out on the GCSA2 index thanks to a backwards searching algorithm, starting from the last character of the read. If and only if the algorithms finds one or more exact-matches on the index, it tries to map the entire read to the graph in the next phase, otherwise the read is rejected. The number of possible initial nodes can be quite large. For this reason we decided an heuristic in order to select the most promising starting nodes. Starting from the first seed node, the mapping phase tries to align the read on the graph exactly, e.g. without gaps. If the alignment includes a nucleotide belonging to an SNP, this alignment represents the best choice, and the alignment of the read is recorded as the best alignment and the process stops. Otherwise, the algorithm tries to map the read from an other initial node, if it exists. Only reads with one or more reference or alternative base of SNPs are included in the output file, and then used in the genotyping process.

The mapping algorithm tries to find a path in the graph, starting from an initial match, that represent the exact alignment of the read. Nodes founds on GCSA2 index must be firstly translated with respect to the IDs present in the variation graph, thanks to the GCSA2 id() query seen previously. The mapping algorithm uses the rc() query on the initial node to decide the direction of the alignment: if rc returns true it looks for a node with an ID lower than the previous one, if rc return false it looks for a node with an ID greater than the previous one. Obviously, this choice is optimal for the biallelic or multiallelic SNPs, but it will not be correct if there are other types of variants which tend to reuse graph nodes. Then, the algorithm tries to align the read exactly, with no gaps, at each node crossed by the search, until the read is completely aligned or a stop condition has been reached. The alignment strategy implements a depth-limited search, where the search space of neighbouring nodes is limited. The graph is explored with a depth first search, that is going as deep as possible following the direction obtained thanks to the initial node. Also, we use two stop conditions on the number of nodes visited. The stop conditions used by the alignment algorithm are the following:

– The maximum number of explored nodes are limited by 2 × *N*, where *N* is the length of the read. This fairly wide choice allows to achieve alignment on graph ensuring finite loop in case of false positives on the initial nodes.
– For each node, only 4 neighboring nodes can be visited.

These values allow to make a safe and faster alignment. Tests with different values were conducted without leading to significant improvements. In general larger values of these parameters will only increase the alignment time, without increasing the number of aligned reads.

**Figure 2:**
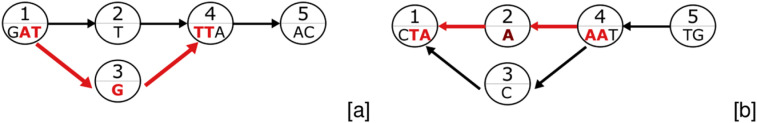
(a) Mapping of the read “ATGTT”, which includes an alternative allele of an SNP, on the variation graph in the forward direction; (b) mapping of read “AAAAT”, which maps to the reference, on the complemented variation graph in the reverse direction.

In Figure 2 we report two examples of reads to graph alignment. In the first example (a) the read “ATGTT” is aligned to the graph in the forward direction, left to right, and it covers the node 3 that is the alternative allele of an SNP. In the second example (b) the read “AAAAT” is aligned in the reverse direction, right to left, on the complemented graph. This read is aligned on the reference genome.

## 3 Implementation

**Figure 3:**
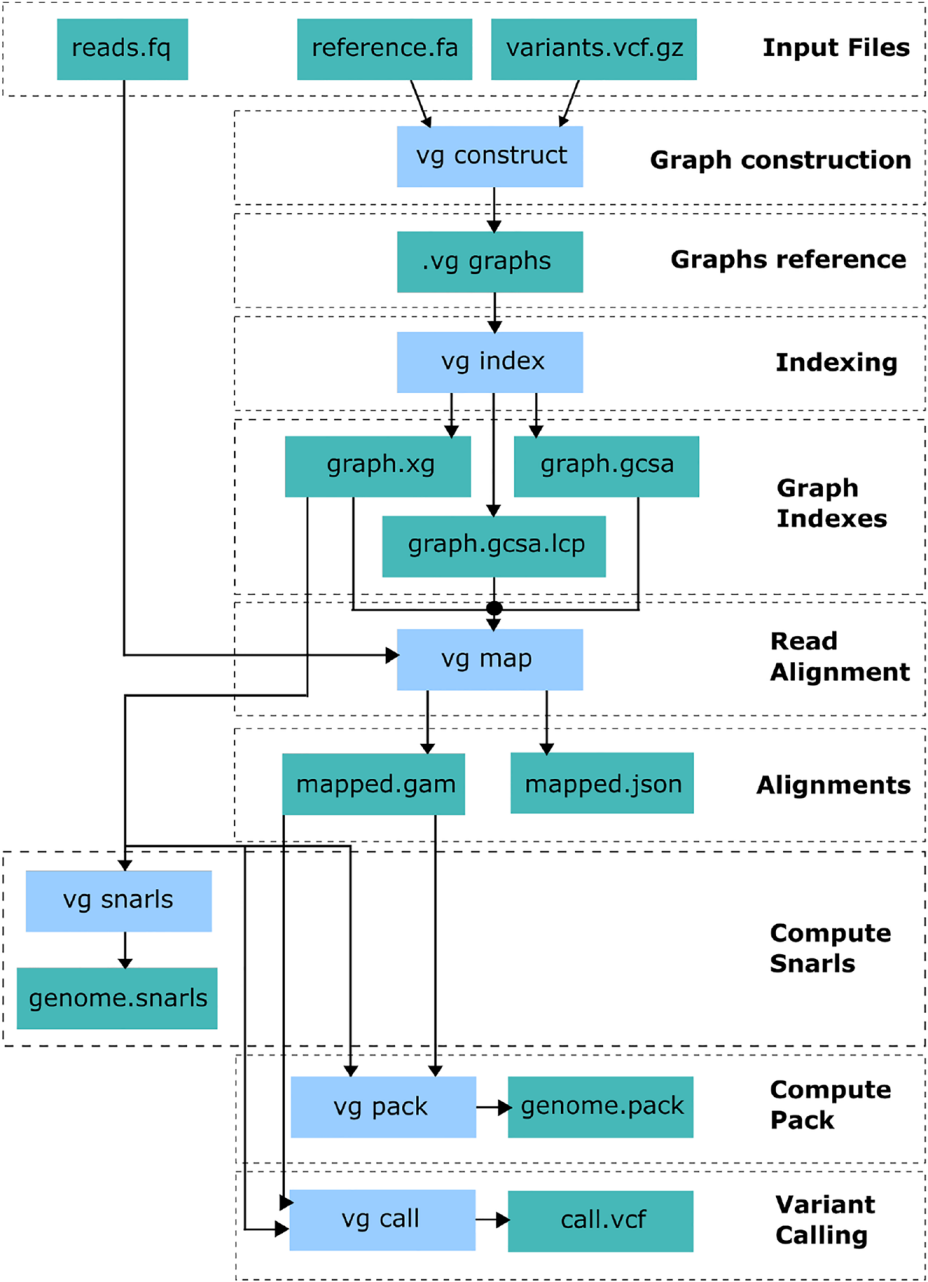
The *vg* pipeline: graph construction, graph indexing, reads mapping, variant calling.

In this section we describe the main differences between the original implementation of *vg* and VG SNP-Aware. A summary of the complete *vg* pipeline is reported in Figure 3. In order to perform the genotyping process *vg* constructs a graph from the reference genome and a set of genomic variations. Then, the graph is indexed and a series of intermediate index files are produced. These indexes are used for reads to graph alignment with the *vg* map command. Finally, the variants are detected using the aligned reads and the variation graph.

The VG SNP-Aware implementation follows the same pipeline. The main differences are in the implementation of the *vg* map command. The new algorithms for initial node search and for mapping have been included in the *vg* map command. In particular it is possible to use the original *vg* mapping or our new version presented here.

The original map command works with few parameters that allow you to set the graph, the index and the dataset of reads. The new map command of VG SNP-Aware includes also the following parameters:

– snp-aware: performs the alignment on the graph with the VG SNP-Aware algorithm described in Section 2.3.
– printsnp: allows to reduce the output size to only reads mapping to reference or alternative base of some SNPs.

To use VG SNP-Aware the snp-aware parameter is required while printsnp is an optional parameter but it is recommended. The -j parameter is mandatory to obtain as output the JSON mapping file. It is possible to use the *vg* view command to switch from JSON to GAM files and vice versa. Here you can find an example of the command options:

– vg map -f reads.fq -x graph.xg -g graph.gcsa –printsnp –snp-aware -j
– vg map -f reads.fq -x graph.xg -g graph.gcsa –snp-aware -j

The pseudocode of VG SNP-Aware alignment of reads to the graph is presented below.

**Algorithm 1**

VG SNP-Aware mapping (input_reads, graph).

**Table:** 

1: **for all** read R ∈ input_reads **do**
2: initial_nodes=find_match(R,graph)
3: **for all** node n ∈ initial_nodes **do**
4: current_node=n
5: max_node_visited= 2 ^*^ length(R)
6: string_to_map= extract_substring(R,n)
7: mapping_direction= find_direction(R,n)
8: neighbour=0
9: aligned_path=current_node
10: **while** (length(string_to_map) > 0 and max_node_visited > 0) **do**
11: next_node=find_next_node(current_node, mapping_direction)
12: match=find_string(string_to_map,next_node)
13: **if** match! =∅ **then**
14: update(string_to_map,match)
15: current_node=next_node
16: neighbour=0
17: add_node(aligned_path, current_node)
18: **else**
19: neighbour++
20: **if** neighbour > 4 **then**
21: exit While;
22: **end if**
23: **end if**
24: max_node_visited = max_node_visited - 1
25: **end while**
26: **if** length(string_to_map) =0 **then**
27: Store_Alignment(R,aligned_path)
28: **if** aligned_path contains a SNP **then**
29: exit for all loop and process next read
30: **end if**
31: **end if**
32: **end for**
33: **end for**

The algorithm starts by searching for an initial match between the read and the nodes in the graph. This search is implemented using the GCSA2 index. Thanks to the GCSA2 functionalities, the algorithm obtains the initial node ids on the original graph. Once the list of initial nodes is populated it is possible to align the read on graph thanks to a depth-limited like search that starts from these nodes. The main idea behind the algorithm proposed here, is to try to align the read at each node crossed by the search, until the read is totally consumed or a stop condition has been reached. The search strategy explores the graph going as deep as possible. The edges of the graph are explored starting from the last node discovered, searching for unexplored edges coming out of it. These candidate nodes are queried for the string_to_map, and if a match is found the search continues along this path. In this case the string_to_map is updated, removing the matching string, and the new node is stored in the alignment path. The search stops if the read is completely aligned or if one of the two stopping conditions is meet. Then, the final alignment path is stored, and if it contains an SNP the search stops and the read is mapped.

## 4 Results

In this section we report the results obtained by the tools *vg* and VG SNP-Aware. For *vg* two possible alignment modes are considered, the first one indicated with a generic *vg* calculates the alignment with possible mismatches, and it is the default option for *vg*. The second one, indicated with *vg* Exact, uses the parameter K to set the length of the reads and to search only for exact alignments. VG SNP-Aware proposed in this work, searches for exact alignments as *vg* Exact, but with a more efficient searching strategy. All tests are based on *vg* version 1.29.0-Sospiro.

### 4.1 Experimental setup

The analysis focuses on two fundamental points, the time taken to complete the alignment and the genotyping process, and the accuracy of the results obtained by the respective tools.

The datasets used in this study are two sets of real reads from the same individual (NA12878) from the 1000 Genomes Project [1]: SRR622461 with a coverage of 6X (40 GB) and SRR622457 with a coverage of 10X (65 GB). These datasets have been widely used for benchmarking in other studies [18–20]. For validation, we used an up-to-date high-quality genotype annotation generated by the Genome in a Bottle Consortium [21]. The GIAB gold standard contains validated genotype information for NA12878, from 14 sequencing datasets with five sequencing technologies, seven read mappers and three different variant callers. The number of validated SNPs for NA12878 in the gold standard is 3.7M. The reference genome used is hg19, also described as Genome Reference Consortium Human Build 37 (GRCh37). For the SNPs dataset we choose dbSNP [8], which contains human single nucleotide variations, and more specifically 11M SNPs biallelic. A summary of the datasets used for the evaluation is reported in Table 1.

**Table 1:** Resources and datasets used for testing.

Dataset	Description
hg19	Reference genome
dbSNP	Biallelic SNPs dataset [8]
NA12878	Gold standard from GIAB [21]
SRR622461 (low coverage)	Dataset of reads with coverage 6×
SRR622457 (high coverage)	Dataset of reads with coverage 10×

To measure the accuracy, we use RTG tools and the SNP list which are also genotyped in the GIAB gold standard. Each evaluation comprises the standard metrics: True Positive, False Positive, False Negative, Precision, Recall and F-Measure. All tests were performed on a 14 lame blade cluster DELL PowerEdge M600 where each lame is equipped with two Intel Xeon E5450 at 3.00 GHz, 16 GB RAM and two 72 GB hard disk in RAID-1 (mirroring).

### 4.2 Mapping reads to a graph: time and memory

In this section we test the requirement of computational resources for all tools. Table 2 reports a summary of the results obtained for the two datasets for all tools. Since the genotyping step is the same for all tools, we report the running times for reads mapping and the genotyping phase separately.

**Table 2:** Time and memory comparison for reads alignment and genotyping for various tools.

Dataset	Software	Mapping min (speedup)	Genotyping min	Total time min (speedup)	Memory GB
Low coverage	*vg*	8685 (38.6×)	120	8804 (28.7×)	47.74
Low coverage	*vg* Exact	1003 (4.45×)	100	1103 (3.1×)	47.74
Low coverage	VG SNP-Aware	225	129	354	45.25
High coverage	*vg*	12,307 (40.6×)	165	12,472 (28.6×)	48.73
High coverage	*vg* Exact	1542 (5.1×)	126	1668 (3.8×)	48.73
High coverage	VG SNP-Aware	303	138	436	51.13

First of all the memory required by all tools is very similar. The memory is mainly dominated by the size of the variation graph, and the corresponding data structures, and it varies only slightly with the size of the input reads. As for the running time the major variations are in the reads mapping phase, where the three tools follow different alignment strategies.

On the low coverage dataset *vg* requires 8685 min to align 179 million reads, that are about 6 days of computation. In the case of *vg* Exact the mapping phase requires 1003 min (16.7 h), whereas VG SNP-Aware only 225 min (3.75 h). Thus, on the low coverage dataset, VG SNP-Aware achieved a speedup of 4.45× with respect to *vg* Exact, and 38.6× with *vg*. If we consider the total running time for mapping and genotyping the speed up of VG SNP-Aware remain 3.1× and 28.7×, w.r.t. to *vg* Exact and *vg* respectively.

It is worth noting that the genotyping phase is the same for all tools. However, for *vg* and *vg* Exact the running time of the genotyping phase is a small fraction of the cost of mapping, whereas in the case of VG SNP-Aware the time for mapping and genotyping are similar. This affects the total speedup. We believe that the current implementation of the genopyting phase can be improved in the case of VG SNP-Aware, considering only SNPs calls like in [22].

If we consider the high coverage dataset, that contains 287 million reads *vg* requires about 8 days of computation for reads mapping, *vg* Exact 1 day, whereas VG SNP-Aware 5 h. As the size of the input dataset grows also the speedup for reads alignment increases, and for the high coverage dataset VG SNP-Aware is 40.6× times faster than *vg*, and 5.1× than *vg* Exact. As for the total running time of mapping and genotyping the speedups on the high coverage dataset are in line with those of the low coverage dataset.

In Table 3 we report the size of the alignment files produced by all tools. All tools produce a graph alignment mapping (GAM) file, that is a binary compressed representation of the reads alignment, very similar to a BAM file. The binary compressed GAM file can be expanded and transformed into a JSON file, both file dimensions are reported in Table 3. Since VG SNP-Aware is designed to detect only SNPs from a given database, e.g. dbSNP [8], we decided to report in the output alignment only the reads that are aligned to the regions of the reference genome that contain some SNPs. This results in a reduction of the output files w.r.t. *vg* Exact of 70 and 66% respectively for the GAM and JSON file. However, it should be noted that a similar strategy can be applied also in the other tools.

**Table 3:** Size of the output alignment file for all datasets and tools.

Dataset	Software	JSON (GB)	GAM (GB)
Low coverage	*vg*	183	17
Low coverage	*vg* Exact	135	14
Low coverage	VG SNP-Aware	46	4.2
High coverage	*vg*	291	27
High coverage	*vg* Exact	211	22
High coverage	VG SNP-Aware	71	6.3

In summary, the most demanding phase is reads alignment and VG SNP-aware is considerably faster, up to 40×, than its competitors.

### 4.3 Genotyping accuracy

In this section we test the genotyping accuracy of all tools on both datasets. The VCF file produced by *vg*, *vg* Exact, VG SNP-Aware are compared with the gold standard [21]. In Table 4 we report the results of all tools in terms of true positive, false positive and false negative. In Table 5 are shown the results in terms of the metrics: precision, sensitivity and F-measure.

**Table 4:** Genotyping results for all tools and datsets: true positive, false positive and false negative.

Dataset	Software	TP	FP	FN
Low coverage	*vg*	1,773,962	95,198	1,917,199
Low coverage	*vg* Exact	1,503,409	73,185	2,187,744
Low coverage	VG SNP-Aware	1,511,408	127,387	2,179,745
High coverage	*vg*	2,141,772	84,743	1,549,384
High coverage	*vg* Exact	1,928,400	57,667	1,762,753
High coverage	VG SNP-Aware	1,935,783	120,288	1,755,370

**Table 5:** Genotyping results for all tools and datsets: precision, sensitivity and F-measure.

Dataset	Software	Precision	Sensitivity	F-measure
Low coverage	*vg*	0.9491	0.4806	0.6381
Low coverage	*vg* Exact	0.9536	0.4073	0.5708
Low coverage	VG SNP-Aware	0.9223	0.4095	0.5671
High coverage	*vg*	0.9619	0.5802	0.7239
High coverage	*vg* Exact	0.9710	0.5224	0.6793
High coverage	VG SNP-Aware	0.9415	0.5244	0.6736

For the low coverage dataset the results of *vg* Exact and VG SNP-Aware are similar, where VG SNP-Aware has an increment of true positive and false positive. Since both find exact alignment, these results are in line with what expected. Note that the number of true positive and false positive depends on the SNPs contained in dbSNPs and how many of them are contained also in the gold standard. This means that VG SNP-Aware identified a greater number of SNP variants present in the dbSNP that are not present in the gold standard. This observation has been reported also in other mapping-free tools like LAVA [22], that uses dbSNP as SNPs reference. The standard *vg*, thanks to the fact that it includes mismatches in the reads alignment, it detects the highest number of true positives.

On the high coverage dataset, thanks to the 10X coverage, the number of detected SNPs is much higher for all tools. Again, *vg* Exact and VG SNP-Aware have similar results in terms of true positive and false negative. As in the previous case, the number of false positive is greater in VG SNP-Aware and this means that the precision values are slightly lower than *vg* and *vg* Exact. However, in terms of F-measure *vg* Exact and VG SNP-Aware have very similar values on both datasets.

In summary, *vg* achieves the best overall performance in terms of the number of detected SNPs, because it allows for inexact reads alignment, however it is extremely slow and it requires days of computation on a cluster. VG SNP-Aware is able to detect SNPs with a slightly lower precision, but with a 40× speedup on reads mapping.

## 5 Conclusions

In this paper we presented VG SNP-Aware an algorithm to speed up the alignment of reads to a variation graph with application to SNP detection. In the case of SNP detection the structure of a variation graph can be exploited for efficiently mapping of reads. VG SNP-Aware is able to reduce the number of accesses to the graph by exploiting the sequential nature of nodes’ IDs. We tested different tools on popular benchmarking datasets for reads genotyping using a variation graph. The results show that VG SNP-Aware is able to align reads to the graph with a speedup of 40× with respect to the most popular tool *vg*. If we consider *vg* Exact the speedup is still high, 5.1×. The precision of VG SNP-Aware is only slightly lower to that of *vg* and in line with *vg* Exact. As the coverage of the dataset grows, the number of aligned reads by VG SNP-Aware increases, thus improving the number of detected SNPs. VG SNP-Aware can also reduce the size of the output alignment files, by reporting only reads mapped near the SNPs. As future direction of investigation it would be interesting to increase the precision by using the information of quality values. Also, VG SNP-Aware could be extended for the detection of other genetic variations such as insertions and deletions. Furthermore, it would be interesting to speedup the genotyping phase of *vg*.
